# NLRP3 inflammasome in atherosclerosis: Mechanisms and targeted therapies

**DOI:** 10.3389/fphar.2024.1430236

**Published:** 2024-07-31

**Authors:** Pengfei Chen, Xia Li

**Affiliations:** ^1^ Marine College, Shandong University, Weihai, China; ^2^ Shandong Kelun Pharmaceutical Co, Ltd., Binzhou, China

**Keywords:** NLRP3 inflammasome, atherosclerosis, mechanisms, targeted therapies, small molecule inhibitors

## Abstract

Atherosclerosis (AS) is the primary pathology behind various cardiovascular diseases and the leading cause of death and disability globally. Recent evidence suggests that AS is a chronic vascular inflammatory disease caused by multiple factors. In this context, the NLRP3 inflammasome, acting as a signal transducer of the immune system, plays a critical role in the onset and progression of AS. The NLRP3 inflammasome is involved in endothelial injury, foam cell formation, and pyroptosis in AS. Therefore, targeting the NLRP3 inflammasome offers a new treatment strategy for AS. This review highlights the latest insights into AS pathogenesis and the pharmacological therapies targeting the NLRP3 inflammasome, focusing on optimal targets for small molecule inhibitors. These insights are valuable for rational drug design and the pharmacological assessment of new targeted NLRP3 inflammasome inhibitors in treating AS.

## 1 Introduction

Atherosclerosis (AS) is a process where the intima of arteries forms plaques and aggravates secondary lesions such as calcification, atheromatous ulcers, and thrombosis (Basatemur et al., 2019). AS affects all large and medium-sized arteries in the human body, and is a critical pathological basis for various cardiovascular diseases (Mensah et al., 2023). Given the severity and complexity of atherosclerotic cardiovascular disease, effective prevention and treatment of AS are important. Previously, AS was considered merely a passive accumulation of cholesterol in vessel walls. However, recent studies suggest that AS is a complex process where chronic inflammation plays a significant role (Arida et al., 2018; Kong et al., 2022; Yang et al., 2023). Genome-wide association studies combined with clonal lineage tracing and clinical trials have demonstrated that the immune response influences the occurrence and development of AS (Wolf and Ley, 2019). Thus, targeting inflammatory pathways could be a viable strategy. Concurrently, the NLRP3 inflammasome, especially in the immune system for detecting pathogens and initiating responses, is crucial in inflammatory response (Shen et al., 2018; Li et al., 2020; Zhang WJ. et al., 2023). However, its abnormal activation can exacerbate AS (Duan et al., 2020). In addition, there is evidence that abnormal activation of NLRP3 inflammatory corpuscle in anthracycline-based drugs such AS mediated role in cardiac toxicity, including cytokine storm and myocardial fibrosis, which undoubtedly exacerbated the AS related degree of the risk of cardiovascular disease (Quagliariello et al., 2021).

At present, the NLRP3 inflammasome has become a focal point as a potential therapeutic target for AS. Research into drugs targeting the NLRP3 inflammasome is ongoing, though no such drugs are yet available. A major barrier is the incomplete understanding of the structure and mechanism of NLRP3. Nevertheless, Recent studies have begun to elucidate these aspects. Notably, in 2019, Sharif et al. reported the cryo-electron microscopic structure of inactive NLRP3 in a complex with NEK7 (Sharif et al., 2019) In 2021, Xiao et al. detailed the cryo-electron microscopy structure of activated NLRP3 oligomer (Xiao et al., 2023), and Dekker et al. described the crystal structure of the NLRP3 NACHT domain, identifying a crucial binding pocket (Dekker et al., 2021) These discoveries provide a clearer picture of the structure and mechanism of NLRP3, aiding the development of small molecule inhibitors targeting NLRP3 inflammasome.

This review will explore the latest advances in the structure and molecular activation of the NLRP3 inflammasome, its role in AS, and the potential target sites for inhibitors.

## 2 Pathogenesis and progression of AS

It is traditionally believed that AS is caused by cholesterol accumulation due to abnormal lipid metabolism, and treatments like statins are based on this theory (Hansson and Hermansson, 2011). This perspective suggests that AS develops from the excessive buildup of lipids, particularly cholesterol, in the arterial wall. Low-density lipoprotein (LDL) crosses the endothelium via transcytosis, is retained by endothelial inflammation, and gradually forms atheromatous plaques. However, recent clinical studies have established that AS is a chronic inflammatory vascular disease influenced by multiple factors. The process of AS involves endothelial cell damage, foam cell accumulation, plaque formation, and related acute clinical lesions, indicating a complexity beyond mere passive lipid accumulation (Kales et al., 2007; Kobiyama and Ley, 2018; Zhuang et al., 2019; Jin et al., 2023). Recent findings also show that statins not only slow cardiovascular disease progression through cholesterol synthesis inhibition but also mitigate T cell-mediated inflammation, contributing to their effectiveness in AS treatment (Shahbaz et al., 2019). This was indirectly supported by the CANTOS trial results published by P.M. Ridker et al. They demonstrated that immune pathways can reduce AS-related cardiovascular disease independently of lipid levels, confirming the significant role of inflammation in AS (Ridker et al., 2017). The progression of AS is marked by changes in endothelial cells, macrophages, and vascular smooth muscle cells (VSMCs), with widespread involvement of the inflammatory response (Maguire et al., 2019). In 1986, Professor Russell Ross from the Washington School of Medicine, USA, first proposed AS an inflammatory disease triggered by an overreaction to injury (Ross, 1986). By 1999, Russell Ross further identified AS a chronic inflammatory disease following observations of monocyte infiltration into developing fatty streaks (Ross, 1999). Recent discoveries have clarified the antigens initiating AS inflammation (Wolf and Ley, 2019), including oxidized LDL (ox-LDL), bacteria, and viruses, which drive the disease by activating various immune cells (Kita et al., 1990; Suciu et al., 2018; Ahmadi et al., 2021; He and Liu, 2023). Additionally, studies indicate that factors like extracellular vesicles from apoptotic cells in AS plaques may further accelerate AS (Xu et al., 2018; Qian et al., 2021).

The initial step in AS development is generally considered to be endothelial cell damage (Yuan et al., 2022). As secretory cells, endothelial cells increase intimal permeability and release substances like vascular cell adhesion molecule-1 (VCAM-1) and monocyte chemoattractant protein-1 (MCP-1) in response to injury or dysfunction (Fu et al., 2018). Leukocytes bind to endothelial cells through adhesion molecules, easily crossing into the vessel wall. Monocytes transform into macrophages under chronic inflammation and sense ox-LDL through various scavenger receptors (SR), with numerous macrophages engulfing ox-LDL to become foam cells (Chistiakov et al., 2017; Xue et al., 2023). Monocyte transformation has long been considered a major source of foam cells. However, recent studies using immunostaining and flow cytometry have shown that VSMCs also form a significant proportion of foam cells in humans and mice, contributing more to autophagy-mediated cholesterol efflux than previously thought (Wang Y. et al., 2019; Robichaud et al., 2022). VSMCs are the main components of the intima of blood vessels. They acquire abnormal proliferation and migration capabilities through phenotypic transformation in vascular remodeling diseases, showing phenotypic diversity and functional complexity in AS (Alencar et al., 2020; Grootaert and Bennett, 2021). When exposed to cholesterol load and inflammatory stimuli, these cells proliferate and migrate to the intima, transforming into a foam cell phenotype with further cholesterol and inflammation stimulation (Deng et al., 2023). However, the exact mechanism of their transformation into foam cell-like structures remains unclear and requires more research.

The formation of foam cells marks the onset of pathological changes typical of AS (Wang D. et al., 2019). Continuous foam cell formation and lipid stripe accumulation in the intima eventually lead to atherosclerotic plaque formation (Xiang et al., 2022). At the lesion site, macrophages and T cells secrete various inflammatory factors, expanding the inflammatory response and attracting more leukocytes to the plaque development. AS progresses, plaques evolve from fatty streaks to intimal dilatation, and VSMCs cover foam cells with secretory fibers to form a fibrous cap, leading to foam cell hypoxic death. Inflammatory cells in fibrous plaques secrete proteases and other mediators that weaken the fibrous cap, potentially causing coronary thrombosis, which may result in infarction and sudden cardiac death (Alonso-Herranz et al., 2023). In addition, advanced AS plaques feature plaque calcification, starting in the inflammatory areas with very small stromal vesicles. Initially, apoptotic foam cells are absorbed by neighboring smooth muscle cells. As the number of apoptotic cells increases, their debris accumulates, forming a necrotic core. The vesicles in this core become nucleation sites for calcium phosphate microcalcifications. Over time, these cells aggregate into larger masses and form calcified lamellar arteries, leading to hemodynamic changes and significant patient risk (Nakahara and Strauss, 2017). It is worth mentioning that neutrophils, one of the most abundant white blood cells in the human body, have recently gained widespread attention for their role in atherosclerosis (AS). Like monocytes, neutrophils are activated by chemokines such as MCP-1 and IL-8 to localize to vascular damage sites (Xue et al., 2019). Recruited neutrophils produce a large amount of reactive oxygen species (ROS) and secrete various inflammatory factors. These actions aggravate endothelial cell damage and dysfunction (Zhang X. et al., 2023). Furthermore, neutrophils can release extracellular chromatin, nucleoprotein, and serine protease. These components form a reticular fiber network, eventually leading to the formation of neutrophil extracellular traps (NETs) (Qi et al., 2017). NETs facilitate direct communication between neutrophils and monocytes, stimulating monocytes to enter AS plaques and modulate inflammatory responses (Josefs et al., 2020). Numerous studies indicate that NETs contribute to the destruction and further development of morphologically distinct plaques in advanced AS stages (Quillard et al., 2015; Franck et al., 2018; Pertiwi et al., 2018). In brief, neutrophils aggravate endothelial dysfunction and promote monocyte recruitment to AS lesions and foam cell formation. In the late stages of AS, neutrophil-derived proteases and ROS lead to unstable plaques (Döring et al., 2017). Therefore, inflammatory events participate in the whole process of AS development.

## 3 NLRP3 inflammasome

**FIGURE 1:**
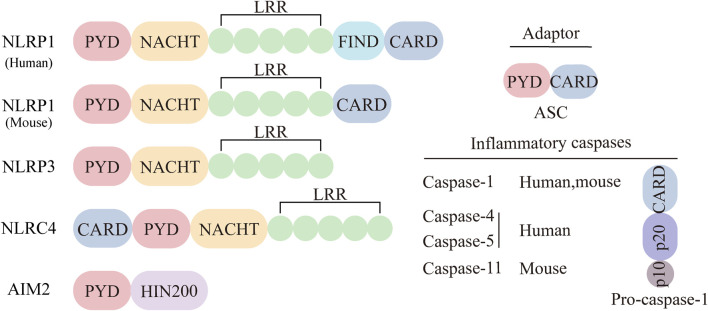
Major categories of inflammasome sensors. The inflammasome can be classical or non-classical inflammasome. The classical inflammasome is composed of three parts: sensing proteins, adaptors, and effectors. The sensing proteins mainly include NLRP1, NLRP3, NLRC4, and AIM2. The adapter ASC consists of two parts, PYD and CARD. The effectors in the classical inflammasome are Pro-Caspase-1.

The term “inflammasome” first appeared in a 2002 article by Tschopp et al., who described it as a complex required for the oligomerization and activation of the proinflammatory protease caspase-1 (Martinon et al., 2002). Further research revealed that the inflammasome is mainly found in innate immune cells that combat pathogens. It detects pathogens and danger signals, initiating inflammatory responses and pyroptosis. (Xu and Núñez, 2023). The typical inflammasome includes a sensor (NLR or ALR receptor), an adapter (apoptosis-associated speck-like protein ASC), and an effector (Pro-Caspase-1) (Ketelut-Carneiro and Fitzgerald, 2020). The non-canonical inflammasome forms when human Pro-Caspase-4/5 (or mouse Pro-Caspase-11) binds to LPS. NLRs are the largest pattern recognition receptors (PRRs) in the innate immune system, essential for detecting pathogens and initiating defense. Besides AIM2, most sensors in inflammasome formation are NLR members. NLR proteins have three conserved domains: an N-terminal interaction domain, a central nucleotide-binding oligomerization domain (NACHT), and a C-terminal leucine-rich repeat (LRR) domain (Inohara and Nuñez, 2001; Harton et al., 2002; Broz and Dixit, 2016; Fu and Wu, 2023). The family divides into NLRP or NLRC, based on the presence of a pyridine domain (PYD) or a caspase activation and recruitment domain (CARD) at the amino terminal, respectively. NLRP1, NLRP3, NLRC4, and AIM2 are well-studied sensors for inflammasome formation (Figure 1). The adapter ASC connects NLRP3 to Pro-Caspase-1, with a PYD at the N-terminal and a CARD at the C-terminal (Sharma and de Alba, 2021). Effector enzymes often have a CARD domain on one side and a specific Caspase on the other. Classical inflammasomes mediate inflammation through Caspase-1, triggering proinflammatory cytokines IL-1β and IL-18. The non-classical inflammasome is a complex of human Pro-Caspase-4/5 (mouse Pro-Caspase-11) and LPS. Activated Caspase-4, 5, and 11 promote pyroptosis by cleaving gasdermin D (GSDMD), which also activates the classical NLRP3 inflammasome to release cytokines (Monie and Bryant, 2015).

### 3.1 Structure and function of NLRP3 inflammasome

**FIGURE 2:**
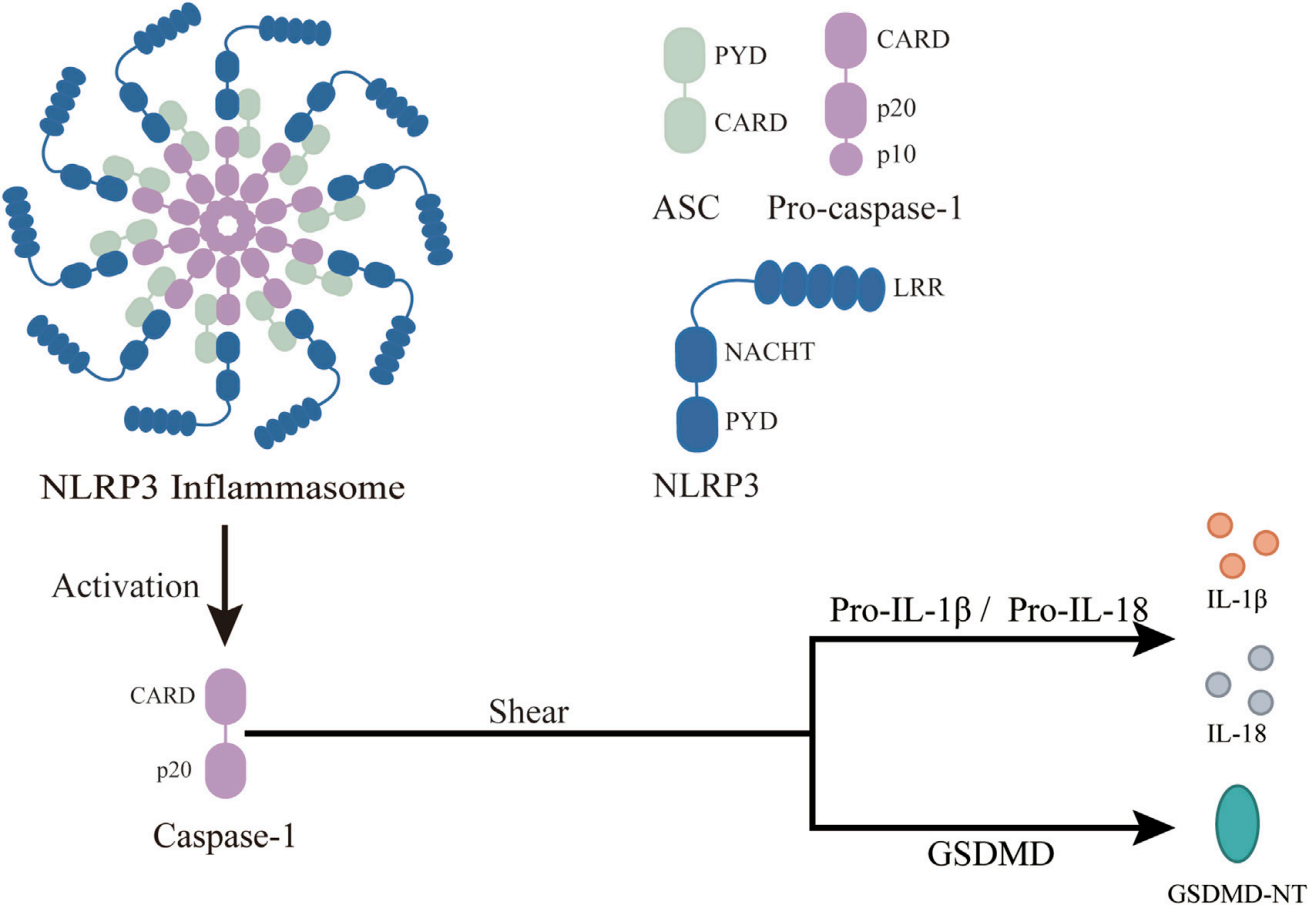
Structure and function of NLRP3 inflammasome. ASC, an apoptosis-related speck-like protein with a molecular weight of 22 kDa, connects NLRP3 and Pro-Caspase-1 in a “dichotomous” form. Pro-Caspase-1 is an effector of NLRP3 inflammasome with a molecular weight of 45 kDa. Once the NLRP3 inflammasome is activated, Pro-Caspase-1 is cleaved to activated Caspase-1. NLRP3, a member of the NOD-like receptor protein family, is composed of three parts: central NACHT, N-terminal PYD, and LRR, which recognize PAMPS and DAMPs. After the inflammasome activation, it changes Pro-Caspase-1 to active Caspase-1, which then covert downstream Pro-IL-1β and Pro-IL-18 to activated IL-1β and IL-18. This leads to a wide range of inflammatory events. In addition, Caspase-1 cleaves GSDMD, leading to the release of GSDMD-NT and pyroptosis.

Among the various inflammasomes, the NLRP3 inflammasome has been most intensively studied since its discovery in 2002 (Shao et al., 2019). The NLRP3 inflammasome is composed of three classes of protein molecules: the sensor NLRP3, the adapter ASC, and the effector Pro-Caspase-1 (Figure 2). The sensor NLRP3, like all NOD-like receptors, functions as a multi-domain signal transducer (Leipe et al., 2004). NLRP3 is divided into three domains: NACHT, PYD and LRR. The NACHT domain binds ATP and mediates ATP-induced NLRP3 oligomerization, while PYD, a conserved protein motif, recruits ASC. Each LRR domain contains 20 to 30 amino acid residues and exhibits self-inhibition and signal recognition capabilities (Hu et al., 2013). When activated, NLRP3 recruits ASC through PYD and induces ASC aggregation into ASC spots, serving as markers of inflammasomes (Lu et al., 2014). The NLRP3-ASC complex then recruits multiple Pro-Caspase-1 and induces its self-cleavage, activating Caspase-1. The structure of NLRP3 inflammasome features “bipolarity” of ASC: the N-terminal PYD of ASC binds to the N-terminal PYD of NLRP3, and the C-terminal CARD binds to the C-terminal CARD of Pro-Caspase-1 through homotypic interaction.

The occurrence of inflammation is primarily mediated by the innate immune system, reflecting the body’s self-regulation after infection or injury (Zhao and Zhao, 2020). At the core of this response is the recognition of pathogen-associated molecular patterns (PAMPS) and damage-associated molecular patterns (DAMPs) by PRR (Amarante-Mendes et al., 2018). When activated, the NLRP3 inflammasome recognizes PAMPS and DAMPs, oligomerizes, and recruits ASC and Pro-Caspase-1, forming the NLRP3 inflammasome complex. The inflammasome then cleaves Pro-Caspase-1 to release Caspase-1, which converts Pro-IL-1β and Pro-IL-18 into active IL-1β and IL-18, triggering a broad range of inflammatory events. Caspase-1 also cleaves gasdermin D (GSDMD), releasing its N-terminal fragment (GSDMD-NT), which translocates to the plasma membrane and forms a 21-nm pore, driving the extracellular release of IL-1β and IL-18 and causing loss of membrane integrity, leading to pyroptosis, a form of inflammatory cell death marked by osmotic swelling and plasma membrane disruption (PMR) (Lamkanfi et al., 2010; Ding et al., 2016; Xia et al., 2021; Santa Cruz Garcia et al., 2022). Additionally, the activation of downstream factors in the NLRP3 inflammasome can enhance the acquired immune response, further boosting the body’s resistance.

### 3.2 Activation of NLRP3 inflammasome

**FIGURE 3:**
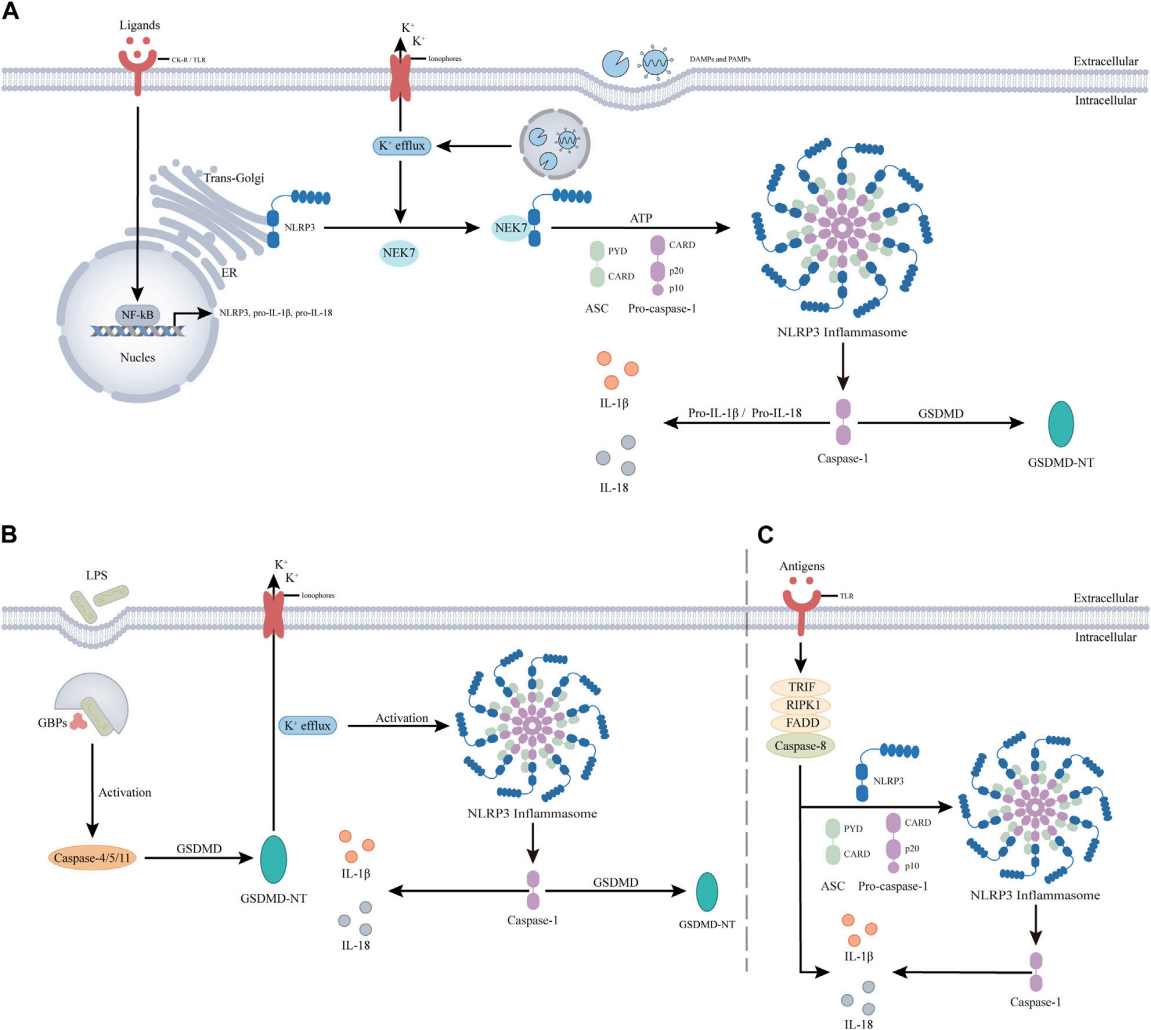
NLRP3 inflammasome activation is divided into three pathways. **(A)**. The classical activation pathway involves two steps, priming and activation. The antigen stimulates cytokine receptor (CK-R) and pattern recognition receptor (PRR), leading to nuclear factor-κb (NF-κB) -mediated transcriptional upregulation of NLRP3, pro-IL-1β, and pro-IL-18. In the activation step, PAMPs and DAMPs are thought to promote the translocation of inactive NLRP3 to subcellular organelles, such as the dispersed trans-Golgi network. NLRP3 is then activated by cellular and molecular events induced by PAMPS or DAMPs, such as K+ efflux, and binds to NIMA-associated kinase 7 (NEK7). NEK7 then interacts with NLRP3 leading to the assembly of the NLRP3 inflammasome through unknown mechanisms, resulting in Caspase-1. Active caspase-1 clears pro-IL-1β and pro-IL-18 into mature forms to induce inflammation and clears GSDMD triggering pyroptosis. **(B)**. The noncanonical activation pathway does not involve a prior priming step. The antigen is detected by guanylate binding proteins (gbps) and induces automatic activation of Caspase-4/5, triggering GSDMD to produce GSDMD-NT. It induces pyroptosis and activates NLRP3 inflammasome by decreasing intracellular K levels. **(C)**. The alternative activation pathway does not contain any features of classical or non-classical NLRP3 inflammasome activation, and antigen stimulation is mediated through the TLR4-TRIF-RIPK1-FADD-CASP8 signaling pathway. The resultant activated NLRP3 participates in caspase-1-mediated IL-1β secretion by an unknown trigger independent of K+ efflux. In addition, in this pathway, Caspase-8 can substitute for Caspase-1 leading to the maturation of IL-1β and IL-18.

NLRP3 inflammasome activation can be categorized into three types (Figure 3): 1) classical, 2) nonclassical, and 3) alternative activation pathways.

The classical pathway involves an initial priming phase and subsequent activation steps. Most NLRP3 inflammasome activation follows this pathway (Vande Walle and Lamkanfi, 2024). In the priming phase, cytokine receptors (CK-R), such as IL-1R, and pattern recognition receptors (PRR), such as Toll-like receptor 4 (TLR4), are stimulated by antigens. This stimulation activates the NF-kB signaling pathway, promoting the upregulation of NLRP3, pro-IL-1β, and pro-IL-18 components. These components form the material basis for subsequent activation, assembly, and function of NLRP3. The priming signal also mediates various post-translational modifications (PTMs) of NLRP3 (Zangiabadi and Abdul-Sater, 2022). For example, LPS exposure downregulates FBXL2-induced ubiquitination degradation of NLRP3 due to the induced level of FBXO3, an E3 ligase targeting FBXL2 (Han et al., 2015). Meanwhile, c-Jun N-terminal kinase (JNK1) phosphorylates NLRP3 S194, essential for deubiquitylation and subsequent inflammasome assembly (Song et al., 2017). In the subsequent activation step, NLRP3 binds to NEK7 upon stimulation by various activators, including PAMPS, DAMPs, and environmental particles and crystals (Shirasuna et al., 2019). The recognition mechanisms of these stimuli are diverse, suggesting NLRP3 senses common signals induced by various stimuli. These triggers lead to critical cellular and molecular events like lysosomal damage, mitochondrial dysfunction, dispersion of trans-Golgi-network (dTGN) and reactive oxygen species (ROS) generation. The molecular events include ion fluxes involving K+, Ca2+, Cl-, and Na+ (Muñoz-Planillo et al., 2013; Yin et al., 2017). Among the various cellular and molecular events, one of the more intriguing phenomena is dTGN, which refers to the breakdown of the trans-Golgi network (TGN) upon different NLRP3 stimulations. Previous studies have demonstrated that NLRP3 is recruited to the dTGN through an ionic bond between its conserved multibase region and the negatively charged phosphatidylinositol-4-phosphate (PtdIns4P) on the dTGN (Chen and Chen, 2018). Subsequently, dTGN serves as a scaffold for NLRP3 aggregation into multiple spots, leading to polymerization of the adaptor protein ASC and subsequent activation of downstream signaling cascades. These findings strongly support a critical role for dTGN in NLRP3 activation, suggesting that targeting dTGH could be a potential strategy for inhibiting NLRP3 inflammasome activity. Importantly, it should be noted that while K+ efflux represents a common signaling pathway associated with agents activating NLRP3, it does not necessarily imply that K+ efflux is indispensable for NLRP3 activation (Jiang et al., 2021).

Nonclassical pathways do not require priming (Moretti et al., 2022). Unlike the Caspase-1 dependence of the classical pathway, the nonclassical pathway depends on Caspase-4/5 in humans and Caspase-11 in mice (Kayagaki et al., 2011). Here, antigens such as LPS initiate the assembly of a cytoplasmic multiprotein scaffold. Guanylate-binding proteins (GBP) coordinate the recruitment and activation of Caspase-4/5/11, leading to the formation of GSDMD-NT, which disrupts the plasma membrane (Santos et al., 2020). This disruption causes K+ efflux and induces pyroptosis, activating NLRP3 and leading to the assembly of the NLRP3 inflammasome (He et al., 2015). Interestingly, K+ efflux in the non-classical pathway can trigger the classical activation pathway.

The alternative pathway lacks classical or nonclassical features, such as ASC spot formation, pyroptosis, or K+ efflux. Activation involves the TLR4-TRIF-RIPK1-FADD-CASP8 pathway, which activates the NLRP3 inflammasome. Caspase-8 can directly mature IL-1β and IL-18, bypassing Caspase-1 (Maelfait et al., 2008; Gaidt et al., 2016; Feltham et al., 2017; Unterberger et al., 2023). Although dependent on Caspase-8, the mechanism of the alternative pathway remains unclear and requires further investigation.

## 4 Role of NLRP3 inflammasome in AS

**FIGURE 4:**
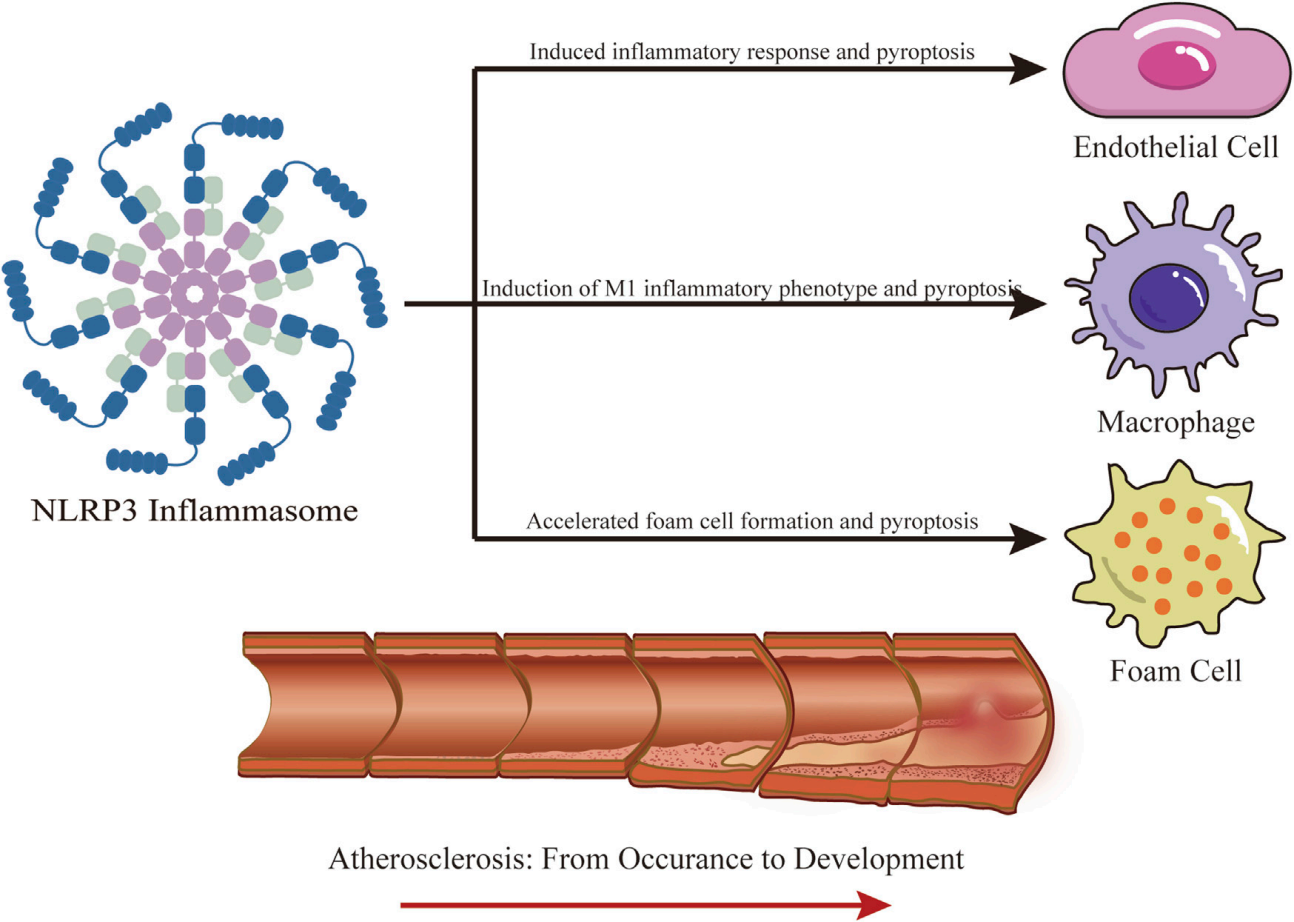
Role of NLRP3 Inflammasome in AS. NLRP3 participates in the occurrence and development of AS, mainly involving three kinds of cells, endothelial cells, macrophages, and foam cells. IL-1β and IL-18 produced by NLRP3 promote the inflammatory response and oxidative stress in endothelial cells, leading to cell injury. In addition, the produced GSDMD-NT causes pyroptosis in endothelial cells; The production of IL-1β and IL-18 by NLRP3 in macrophages creates an inflammatory environment, inducing their differentiation into M1 inflammatory phenotype, and pyroptosis. NLRP3 is widely involved in the formation of foam cells, especially inducing the secretion of high mobility group box-1 protein (HMGB1) in VSMCs, promoting their foamy transformation, pyroptosis of foam cells, and the release of DAMPs and other contents.

Studies have shown that damage to endothelial cells, inflammatory response, and abnormal lipid metabolism play crucial roles in AS. The NLRP3 inflammasome, a key component of the immune system, is involved in both endothelial cell damage in AS and serves as a bridge between lipid metabolism and inflammatory response (Meyers and Zhu, 2020). These connections are clear at the cellular level (Figure 4). Previously, we mentioned that AS primarily involves three types of cells: endothelial cells, macrophages, and foam cells. Excessive activation of NLRP3 inflammasomes can directly damage endothelial cells (Broz and Dixit, 2016). Moreover, the NLRP3 inflammasome is highly expressed in AS plaques, mainly in macrophages and foam cells (Paramel et al., 2016). We therefore now discuss the specific role of the NLRP3 inflammasome in AS, focusing on endothelial cells, macrophages, and foam cells.

Activation of NLRP3 inflammasomes triggers the secretion of active IL-1β and IL-18, which significantly increase inflammatory response and oxidative stress in endothelial cells (Syed et al., 2020). IL-1β activation also enhances the secretion of adhesion molecules and chemokines in the intima, further aggravating inflammation in endothelial cells. Excessive inflammation and oxidative stress promote endothelial dysfunction, which in turn promotes each other in subsequent processes (Grebe et al., 2018). Additionally, GSDMD-NT, a cleavage product of GSDMD mediated by NLRP3, causes endothelial cell pyroptosis, characterized by cell membrane breakdown, cell lysis, and the release of inflammatory contents including IL-1β, IL-18, and HMGB1 (high-mobility group box-1 protein) (Wolfson et al., 2011). HMGB1 also activates the NF-κB pathway and increases the secretion of inflammatory mediators, indirectly causing endothelial barrier dysfunction. Pyroptosis mediated by NLRP3 has been identified as a potential cause of endothelial cell death (Chen et al., 2019). Various vasoactive substances, such as endothelin-1 (ET-1), nitric oxide (NO), prostanoid, angiotensin 2 (Ang II), vascular cell adhesion molecule-1 (VCAM-1), and intercellular adhesion molecule-1 (ICAM-1), produced during endothelial damage, play crucial roles in endothelial barrier dysfunction and contribute materially to the development of AS (Gao and Galis, 2021).

Macrophages are present in the early stages of AS and play a crucial role in the local inflammatory response and plaque rupture (Khoury et al., 2021). Initially, monocytes gather in the arterial wall and bind to endothelial cells, driven by endothelial chemokines and cell adhesion molecules (Sager et al., 2016). As monocytes migrate to the ductus arteriosus, they develop into macrophages depending on the local microenvironment. Macrophages are classified into two phenotypes: M1 inflammatory phenotype and M2 anti-inflammatory phenotype. The M1 macrophages produce proinflammatory cytokines and reactive oxygen and nitrogen species. In contrast, the M2 phenotype suppresses inflammation and promotes tissue repair (Jinnouchi et al., 2020). With increased cholesterol transport activity in injured macrophages, these cells ingest a large amount of LDL oxidized by their free radicals and attempt to digest it. Macrophage scavenger receptors facilitate the storage of oxidized cholesterol, giving the macrophages a typical “foam” appearance (Liu et al., 2021). NLRP3 is activated extensively by antigens such as ox-LDL and cholesterol crystals, which recruit ASC and Pro-Caspase-1 to assemble the NLRP3 inflammasome, triggering inflammatory responses and inducing macrophages to differentiate into the M1 phenotype. This process contributes to the development of AS (Vande Walle and Lamkanfi, 2024).

Foam cells play a key role in AS lesions (Wang et al., 2018). These cells originate from two sources: the uptake of ox-LDL by macrophages and the transformation of VSMCs into a foamy phenotype (Dinarello et al., 2012; Vengrenyuk et al., 2015). Notably, 60%–70% of foam cells in AS lesions originate from VSMCs, and not from macrophages. While foam cell production is beneficial early in AS, in advanced lesions, these cells die and release DAMPs into the extracellular space (Tabas and Bornfeldt, 2016; Martinet et al., 2019). Activation of the NLRP3 inflammasome in VSMCs leads to the secretion of HMGB1, which downregulates liver X receptor α and ATP-binding cassette transporter expression via the receptor for advanced glycation end products, playing a significant role in the development of vascular smooth muscle cell-like foam cells and atherosclerosis (Burger et al., 2021).

## 5 Pharmacological intervention of NLRP3 inflammasome in AS

The NLRP3 inflammasome plays a crucial role in the onset and progression of AS and serves as an excellent target for pharmacological intervention. Even with the worldwide COVID-19 epidemic of the past few years, targeting the NLRP3 inflammasome has been a strategy to prevent AS-based cardiovascular outcomes in patients with SARS-CoV-2 infection (Quagliariello et al., 2020). In recent years, efforts to inhibit the NLRP3 inflammasome have stimulated the discovery and development of various NLRP3 inhibitors. These efforts have primarily focused on regulating NLRP3 protein transcription, translation, and activation. Small molecule inhibitors are considered a direct and ideal approach for treating AS.

### 5.1 Regulation of NLRP3 protein transcription and translation

#### 5.1.1 MicroRNAs (miRNAs)

MiRNAs are endogenous non-coding RNAs, about 22 nucleotides long, that fine-tune gene expression post-transcriptionally (Naqvi and Sarwat, 2022). MiRNAs regulate macrophage and VSMC activity, endocrine function, and LDL metabolism, playing a key role in AS development (Aryal et al., 2014). Recent preclinical studies have highlighted mir-223-3p as particularly significant in controlling NLRP3 inflammasome levels (Bauernfeind et al., 2012). Mir-223-3p reduces NLRP3 expression by targeting its mRNA, blocking assembly and activation of the NLRP3 inflammasome, and diminishing AS inflammation. Additionally, miRNA-9 suppresses the NLRP3 inflammasome by regulating the JAK1-STAT1 pathway (Wang et al., 2017). Mir-183 targets TXNIP to lessen inflammation caused by the TXNIP-NLRP3 inflammasome (Miao et al., 2020). miRNA-30c-5p prevents endothelial cell pyroptosis induced by the NLRP3 inflammasome through the downregulation of FOXO3-mediated signaling (Li et al., 2018). Given the critical role of miRNAs in AS progression, developing inhibitors that target NLRP3 inflammasome regulation by miRNAs appears promising. “However, given the miRNA may also have the function of the regulation of a variety of gene transcription. In the future, in the development of relevant inhibitors need to pay special attention to the specificity of the possible problems and the resulting effects.”

#### 5.1.2 Regulation of ubiquitination

Ubiquitination is a form of post-translational modification where ubiquitin proteins are coupled to substrate proteins. Ubiquitin (Ub), a 76-amino acid protein, typically binds to lysine residues of substrate proteins via isopeptide bonds (Komander and Rape, 2012). The NLRP3 inflammasome is vital for both innate and adaptive immunity. Proper expression levels are crucial for bodily self-protection (Zhang et al., 2021; Papadakos et al., 2022). Excessive NLRP3 protein upregulation and accumulation can lead to excessive NLRP3 inflammasome assembly (Jiang et al., 2021). Controlling NLRP3 protein levels, the central component of the NLRP3 inflammasome, maintains NLRP3 in an ineffective ubiquitination state, preventing unnecessary activation (Chen et al., 2020). Ubiquitination involves linking three enzymes: ubiquitin-activating enzyme E1, ubiquitin-conjugating enzyme E2, and ubiquitin-ligase enzyme E3. E1 catalyzes ATP-dependent ubiquitin activation and forms a thioester bond with its active cysteine at the C-terminal. This cysteine then transfers Ub to the cysteine binding site of E2, and E3 transfers Ub to the substrate protein (Komander and Rape, 2012; Moynagh, 2014). E3 specifically targets substrate proteins. Targeting these ligases may offer a therapeutic strategy for treating NLRP3 inflammasome-related inflammatory diseases.

Ubiquitination of NLRP3, with K48-linked or K63-linked ubiquitin chains, negatively regulates inflammasome activation. It targets NLRP3 for proteasomal degradation or prevents protein-protein interactions (Akbal et al., 2022). Studies indicate that of the three enzymes required for ubiquitination, E3 ligase primarily relates to NLRP3 protein regulation (Lopez-Castejon, 2020). To date, several E3 ligases have been identified that downregulate NLRP3, including SCF-FBXL2, TRIM31, and Cbl-b. SCF-FBXL2 targets K689 and initiates NLRP3 protein degradation, maintaining a low level of NLRP3 protein in living cells (Han et al., 2015). TRIM31, with its N-end RING structure domain, directly interacts with the NLRP3 PYD structure domain and induces numerous K48 ubiquitin connections. This process leads to NLRP3 proteasome degradation, activating and inactivating cells (Song et al., 2016). Cbl-b, via its ubiquitin-associated domain (UBA), binds to the K63-linked ubiquitin chain in the LRR domain of NLRP3. This binding causes ubiquitination of the K48 link at K496 in the NLRP3NBD domain, inducing proteasomal degradation (Tang et al., 2020). Evidence suggests that NLRP3 ubiquitination and degradation also occur in the nervous system, where dopamine, as an endogenous inhibitor, relies on March7-mediated K48 polyubiquitination in the LRR domain (Yan et al., 2015). Moreover, deubiquitination is considered necessary for the complete activation of NLRP3. This modification may facilitate rapid NLRP3 activation shortly after triggering (Juliana et al., 2012). Ubiquitin-specific peptidases USP7 and USP47 can diminish ASC speck formation and inflammasome activation (Palazón-Riquelme et al., 2018). However, the exact mechanism remains potentially unclear. Thus, promoting the role of ubiquitin E3 ligase on NLRP3 or inhibiting ubiquitin peptidase activity may serve as potential treatment strategies.

#### 5.1.3 Regulation of phosphorylation

Phosphorylation, a common post-translational modification in humans, affects over 30% of cellular proteins. It involves protein kinases transferring the γ-phosphate group from ATP or GTP to amino acids like serine, threonine, and tyrosine. The reverse process involves protein phosphatases removing these phosphate groups. The interplay between these enzymes, along with associated energy changes, makes phosphorylation a key regulatory mechanism for many physiological activities. It regulates inflammasome activation by targeting various inflammatory components, including NLRP3, Pyrin, NLRC4, IFI16, ASC, and Caspase-1. Numerous phosphorylation events control the initiation, assembly, localization, and degradation of NLRP3 inflammasomes (Gong et al., 2018). As most NLRP3 phosphorylation studies are *in vitro*, further *in vivo* studies in relevant models are needed to understand mechanisms relevant to endogenous activation (Sandall and MacDonald, 2019). A deeper understanding of NLRP3 phosphorylation could lead to new dephosphorylation targets for treating AS. Characterization of critical NLRP3 phosphorylation events includes c-Jun N-terminal kinase (JNK) at the Jun Ser198, protein kinase D (PKD), and protein kinase A (PKA) at Ser295, along with an unidentified kinase at Tyr861 (Mortimer et al., 2016; Zhang et al., 2017; Chu et al., 2021). Instead of receptor protein tyrosine phosphatase 22 (PTPN22) mediating NLRP3 phosphorylation (Spalinger et al., 2023), PKA phosphorylation at the Ser295 locus and PKD dependency are considered highly significant. In contrast, Ser295 phosphorylation can significantly reduce ATP enzyme activity, a feature unique to NLRP3 (Sandall and MacDonald, 2019). This is an intriguing research area in NLRP3 phosphorylation regulation. While most NLRP3 phosphorylation studies are *in vitro*, further *in vivo* research is necessary for validation. The regulation of the NLRP3 inflammasome by phosphorylation has introduced a promising avenue for treating AS-related diseases.

### 5.2 Regulation of the initiation and activation of NLRP3 inflammasome

#### 5.2.1 Autophagy inducers

Autophagy is a metabolic pathway that is essential for maintaining cell homeostasis. There are two types of autophagy: non-selective and selective. Non-selective autophagy allows cells to recycle nutrients in energy-limited environments. In contrast, selective autophagy primarily removes intracellular organelles to preserve cellular structure, such as mitochondrial autophagy is selective autophagy (Cao et al., 2019). Autophagy dysfunction can trigger diseases with hyperinflammation and hyperactivation of the NLRP3 inflammasome and is a key regulator of this inflammasome (Biasizzo and Kopitar-Jerala, 2020). Recently, the importance of autophagy has increased in research on AS. Studies have shown that selective autophagy can suppress NLRP3 inflammasome activation (Zhu and Liu, 2022). Dysfunctional autophagy can increase the secretion of NLRP3 inflammasome-related inflammatory cytokines (Razani et al., 2012). This suggests a new therapeutic avenue for AS. Several autophagy regulators have been identified that inhibit the NLRP3 inflammasome, thus mitigating AS. For example, resveratrol, a plant polyphenol, prevents mitochondrial damage in macrophages, reducing NLRP3 inflammasome activation and resulting in IL-1β secretion and pyroptosis (Chang et al., 2015). Melatonin reduces the size and vulnerability of AS plaques by inducing mitophagy, which suppresses NLRP3 inflammasome activity and limits the release of mitochondria-derived DAMP (Ma et al., 2018). This indicates that selective autophagy inducers, especially mitophagy inducers, have broad utility in controlling the NLRP3 inflammasome and ameliorating AS.

#### 5.2.2 Small molecule inhibitor (SMIs)

**FIGURE 5:**
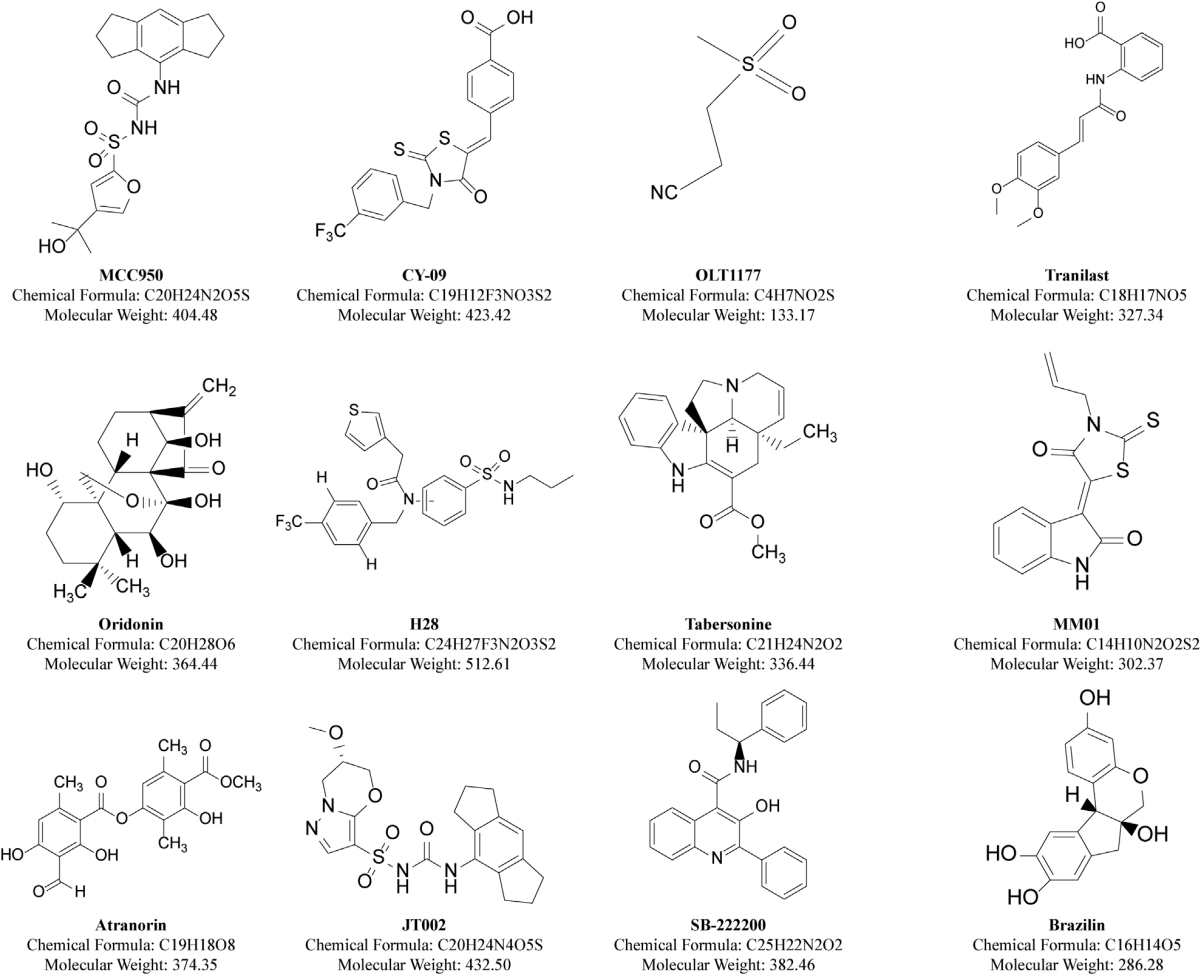
Structures of classical and recent small molecule inhibitors of NLRP inflammasomes.

Inhibiting the NLRP3 protein with SMIs is currently the most straightforward and logical approach to reduce NLRP3 inflammasome-associated inflammation. SMIs are typically more cost-effective than protein-based biologics and can be orally administered, leading to better adherence and fewer side effects. They also provided better efficacy and improved pharmacokinetics in inhibiting the NLRP3 inflammasome. Classical and recent NLRP inflammasome-specific SMIs are summarized (Figure 5; Table 1).

**TABLE 1:** Specific small molecule inhibitors and their inhibitory mechanisms.

Name of compound	Direct target/Potency level (*in vitro*)	Mechanism of inhibition	R&d (for the NLRP3 inflammasome)	References
MCC950	NACHT/nm	Specific inhibition of ATP hydrolysis sites in NACHT blocks NLRP3 activation	Phase II clinical, and then terminated	Coll et al. (2015) Li et al. (2022)
CY-09	NACHT/μM	Specific inhibition of the ATP-binding site in NACHT blocks NLRP3 activation	Preclinical study	Jiang et al. (2017)
OLT1177	NACHT/μM	Specific inhibition of atpase activity in NACHT blocks NLRP3 activation	Phase 3 clinical trial	Marchetti et al. (2018)
Tranilast	NACHT/μM	It binds to NACHT and blocks NLRP3 oligomerization	Preclinical study	Huang et al. (2018)
Oridonin	NACHT/μM	It blocks the NLRP3-Nek7 interaction with cysteine residue 279 within the NACHT domain	Preclinical study	He et al. (2018)
H28	NACHT/μM	Specific inhibition of the ATP-binding site in NACHT blocks NLRP3 activation	Preclinical study	Huang et al. (2024)
Tabersonine	NACHT/μM	Specific inhibition of atpase activity in NACHT blocks NLRP3 activation	Preclinical study	Xu et al. (2023)
MM01	ASC/μM	Binding to the surface of ASC CARD blocks the function of ASC.	Preclinical study	Soriano-Teruel et al. (2021)
Atranorin	ASC/μM	It binds directly to ASC.	Preclinical study	Wang et al. (2023)
JT002	Indeterminacy/nM	Selective inhibition of NLRP3 inflammasome function	Preclinical study	Ambrus-Aikelin et al. (2023)
SB-222200	Indeterminacy/μM	NEK7 binds to NLRP3 protein to block the interaction between nek7 and NLRP3	Preclinical study	Zhou et al. (2023)
Brazilin	NACHT/μM	NACHT was specifically inhibited to block NLRP3 activation	Preclinical study	McMahon et al. (2024)

##### 5.2.2.1 MCC950

MCC950 includes a group of sulfonylurea-containing compounds such as glyburide. Originally developed by Pfizer as CRID3D, it was renamed following an intensive study by Coll et al. (2022). In 2009, Lamkanfi et al. showed that glyburide specifically inhibited NLRP3 signaling (Lamkanfi et al., 2009). MCC950 interacts with the Walker B motif in the NACHT domain of NLRP3 through both covalent and non-covalent interactions. This interaction inhibits ATP hydrolysis and prevents oligomerization of the NLRP3 inflammasome (Masters et al., 2010; Coll et al., 2019). MCC950 also reverses the active conformation of NLRP3 (Tapia-Abellán et al., 2019). MCC950 inhibits both classical and nonclassical activation of NLRP3 by all known activators and is highly specific to the NLRP3 inflammasome. It does not affect the AIM2, NLRC4, and NLRP1 inflammasomes or the TLR pathway activation. However, in a study of phase II clinical trials in rheumatoid arthritis, MCC950 test for liver toxicity and make patients serum enzyme levels show the hepatotoxicity and be terminated (Mangan et al., 2018). Researchers have speculated that this may be related to the metabolically active furan moiety and the very high clinical dose used in the trial (Song et al., 2017). In order to solve this problem, Professor Qin Chong and Associate Professor Jiang Yuqi from Ocean University of China optimized the furoan group of MCC950 based on the binding mode of MCC950 and NLRP3 protein through rational drug design, and found a safe and effective novel NLRP3 inflammasome inhibitor N14. N14 is significantly superior to MCC950 in safety and is more effective than MCC950 in animal disease models of nonalcoholic steatohepatitis, septic shock, and colitis (Li et al., 2023). All this evidence suggests that this hepatotoxicity may be an “off-target” side effect rather than a direct consequence of MCC950 targeting the NLRP3 inflammasome. Despite this, MCC950 remains a potent NLRP3 inflammasome inhibitor and is commonly used to study NLRP3 inhibition mechanisms and assess NLRP3 function in animal disease models. Derivatives of MCC950, mainly N-cyanosulfonylurea and dilute sulfonylurea derivatives, are still under clinical research (Agarwal et al., 2020). Researchers note that MCC950 derivatives might share similar NLRP3 inhibitory effects and mechanisms with MCC950 (Lamkanfi et al., 2009). Current investigations continue to explore the stable targeted inhibition by MCC950 derivatives, emphasizing the sulfonylurea skeleton’s significance in studying NLRP3 small molecule inhibitors.

##### 5.2.2.2 CY-09

CY-09, an analog of the cystic fibrosis transmembrane conductance regulator (CFTR) channel inhibitor C172, inhibits Casp1 activation and IL-1β secretion in LPS-stimulated BMDM at doses of 1–10 μM. It targets monosodium urate, nigericin, and ATP-induced activation. Further studies showed that Walker B motif of NLRP3 NACHT domain was a specific ATP-binding site, which effectively inhibited ATPase activity, while CY-09 targeted Walker B motif. This prevents NLRP3 oligomerization and disrupts the assembly of the NLRP3 inflammasome (Jiang et al., 2017). Additionally, Yu et al. discovered through molecular docking that CY-09 blocks the binding of NIMA-associated kinase 7 (NEK7) to NLRP3, a critical step in NLRP3 inflammasome activation (Wang et al., 2022). CY-09 does not affect other inflammasomes or proinflammatory signaling pathways such as NF-kB, highlighting its specific inhibition of NLRP3.

##### 5.2.2.3 OLT1177

OLT1177, also known as Dapansutrile, is a beta sulfonyl nitrile compound first identified for arthritis topical treatment. In 2019, Fernandez et al. (Sánchez-Fernández et al., 2019) reported that OLT1177 orally exerts a powerful anti-inflammatory effect in experimental allergic encephalomyelitis (EAE) mice. It inhibits the NLRP3 inflammasome, playing a beneficial role in prevention and treatment. Mechanistically, it inhibits NLRP3-ASC interaction by blocking the NACHT ATPase activity of NLRP3, affecting inflammasome assembly; however, the specific binding site remains unclear (Toldo et al., 2019). Although OLT1177 reduced IL-1β and IL-18 release after activating the classical and non-classical NLRP3 inflammasome, it did not affect NLRC4 and AIM2 inflammasome activation. This suggests that OLT1177 could be a novel treatment for AS, specifically by inhibiting the NLRP3 inflammasome.

##### 5.2.2.4 Tranilast

Tranilast, a tryptophan metabolite analog, is a clinical anti-allergic drug with proven efficacy and safety for asthma and allergic dermatitis (Jin et al., 1998). Tranilast inhibited NLRP3 inflammasome activation but did not affect AIM2 or NLRC4 inflammasome activation in macrophages. Mechanistically, Tranilast binds directly to the NACHT domain of NLRP3 and inhibits its assembly by blocking NLRP3 oligomerization; the specific binding site is still under investigation (Huang et al., 2018). Additionally, Tranilast effectively enhanced NLRP3 ubiquitination and reduced vascular inflammation and atherosclerosis in low-density lipoprotein receptor and apolipoprotein E-deficient mice (Chen et al., 2020). As an anti-allergic drug, Tranilast shows promise for anti-inflammatory drug research and development, particularly for anti-AS.

##### 5.2.2.5 Oridonin

Oridonin (Ori) is derived from the Carica genus of the Labiaceae family. Its main component is ent-kau-renediterpenoid, a kaurane-type organic compound widely used in traditional Chinese medicine (Kuo et al., 2014). It inhibits the NLRP3 inflammasome by forming a covalent bond with cysteine residue 279 within the NACHT domain, blocking the NLRP3-Nek7 interaction (He et al., 2018). NEK7 is essential for NLRP3 inflammasome activation (Sharif et al., 2019). Additionally, Ori inhibited the NF-κB and MAPK pathways, reducing the release of NLRP3 inflammasome-independent inflammatory factors (Abu-Ghefreh et al., 2009). Given the inhibitory effect of Ori on the NLRP3 inflammasome and other pathways, it holds potential as an NLRP3 inflammasome inhibitor for AS treatment.

##### 5.2.2.6 H28

H28 was developed through the design and synthesis of novel biphenyl sulfonamide NLRP3 inflammasome inhibitors. It demonstrated a strong inhibitory effect on the NLRP3 inflammasome with an IC50 value of 0.57 μM against the mouse macrophage J774A.1 line. In molecular docking studies, H28 stably bound to the ADP active site in the NACHT domain of NLRP3, identifying a specific mechanism for its action (Huang et al., 2024). *In vitro*, H28 selectively inhibited NLRP3 inflammasome activation without affecting NLRC4 or AIM2 inflammasomes. Additionally, H28 has favorable pharmacokinetic properties, making it a promising candidate for drug development as an NLRP3 inflammasome inhibitor.

##### 5.2.2.7 Tabersonine

Tabersonine, a natural product derived from Catharanthus roseus, is used as an antineoplastic agent. It inhibits NLRP3-mediated IL-1β production with an IC50 of 0.71 μM in mouse-derived macrophages. Studies also reveal that Tabersonine reduces ATPase activity by binding to the NLRP3 NACHT domain; the binding site is near the ATP hydrolysis motif (Huang et al., 2024). Thus, it is a natural NLRP3 inhibitor and a lead for designing new inhibitors.

##### 5.2.2.8 MM01

MM01 is a novel broad-spectrum inflammasome inhibitor useful for treating diseases with inflammasome involvement. It prevents procaspase-1 activation *in vitro* and various cell types in micromolar quantities. *In vivo*, MM01 reduces inflammation in a mouse peritonitis model (Soriano-Teruel et al., 2021). Structural modeling shows it binds to the ASC CARD, blocking necessary interactions for inflammasome assembly and activation. Continued study of MM01 as a small molecule NLRP3 inhibitor is crucial.

##### 5.2.2.9 Atranorin

Atranorin, from lichens, possesses anti-inflammatory properties, reducing nitric oxide and COX-2 levels (Bugni et al., 2009; Mendili et al., 2022). It prevents NLRP3 inflammasome activation in macrophages and dendritic cells by binding to the ASC protein, although the exact binding site is unknown. Atranorin emerges as a promising NLRP3 inhibitor targeting ASC proteins.

##### 5.2.2.10 JT002

Recent findings highlight JT002 as an effective inhibitor of NLRP3 inflammasome-mediated responses, reducing inflammatory factors and pyroptosis in both canonical and non-canonical NLRP3 inflammasome activation pathways. Although not yet proven to bind directly to NLRP3, JT002 is identified as a selective and potent inhibitor (Ambrus-Aikelin et al., 2023).

##### 5.2.2.11 SB-222200

A recent study identified SB-222200 as a promising novel direct NLRP3 inhibitor using drug affinity responsive target stability assay (DARTS), cellular thermal shift assay (CETSA), and surface plasmon resonance (SPR) analysis (Zhou et al., 2023). Mechanistically, SB-222200 inhibits the assembly of NLRP3 inflammasome by directly binding to NLRP3 protein, blocking the interaction between NEK7 and NLRP3 and the oligomerization of NLRP3. These findings suggest SB-222200 potential as a lead compound in NLRP3 inhibitor development.

##### 5.2.2.12 Brazilin

Brazilin, a natural small molecular isoflavonoid, found in several tree species like Caesalpinia echinate and Haematoxylum brasiletto, has been used traditionally for various ailments including skin diseases, diabetes, fever, and cancer (Nava-Tapia et al., 2022). Recent research indicates Brazilin targets an unspecified site in the NLRP3 NACHT domain, enhancing NRF2 signaling and inhibiting NLRP3 activation (McMahon et al., 2024).

## 6 Small molecule inhibitor binding site analysis

The NLRP3 inflammasome, consisting of NLRP3, ASC, and Pro-Caspase-1, provides various binding pockets for small molecule inhibitors. This variation is evident in both classic and recent inhibitors. To further explore optimal binding sites, a detailed analysis of the NLRP3 inflammasome components will follow.

### 6.1 Targeting NLRP3

**FIGURE 6:**
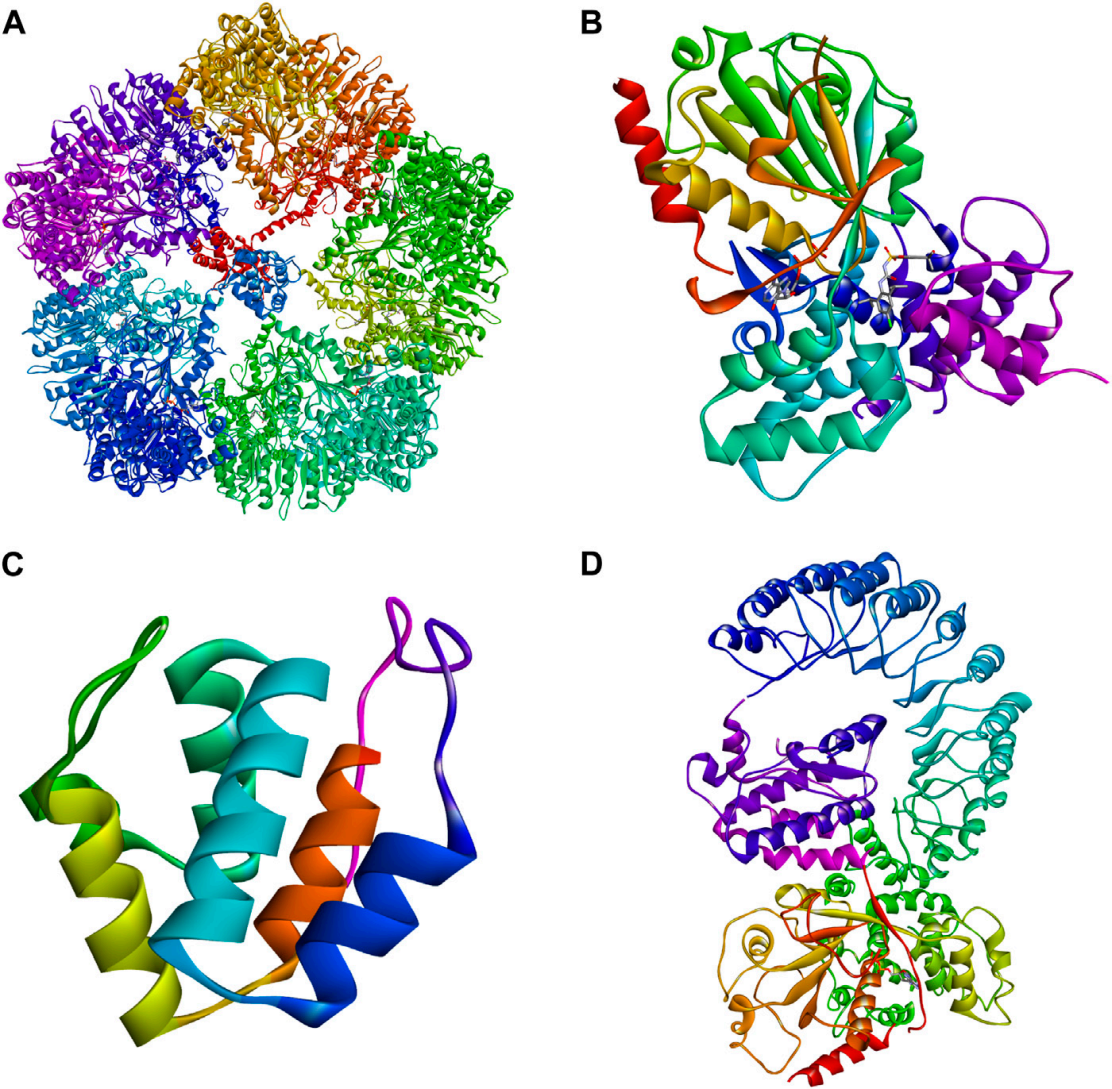
Three-dimensional structures of NLRP3. **(A)** Cryo-EM structure of inactive full-length human NLRP3 (PDB: 7PZC) (Hochheiser et al., 2022). **(B)** Crystal structure of human NACHT (PDB: 7ALV) (Dekker et al., 2021). **(C)** The crystal structure of human PYD (PDB: 3QF2). **(D)** Human with NEK7 NLRP3 NACHT - freeze electron microscopic structure of LRR (PDB: 6NPY) (Sharif et al., 2019).

**FIGURE 7:**
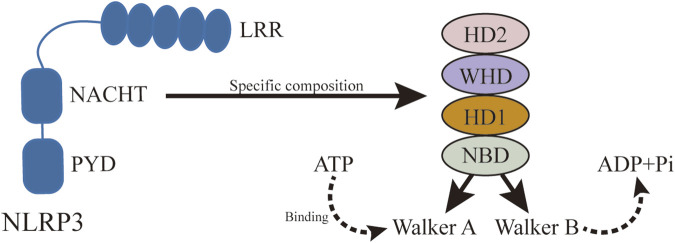
Specific structure of NACHT. NLRP3 activation is ATP-dependent, and as a member of STAND, the central domain of NLRP3, NACHT, plays an important role in ATP binding and hydrolysis. NACHT consists of NBD, HD1, WH, and HD2, in which ATP hydrolysis and binding motifs are located.

Although NLRP3 includes three parts: the central NACHT, the N-terminal PYD, and the LRR (Figure 6), the focus here is on NACHT’s potential drug-binding sites due to extensive research on its inhibitors. Hughes (Hughes, 2006) compared the gene sequences of 14 nod proteins, revealing a conserved ATP-binding motif within the NACHT domain, classifying NLRP3 as a multidomain signal transduction ATPase (STAND) (Leipe et al., 2004). STAND proteins feature a conserved core with ATPase activity and key domains for stimulus sensing and signaling (Hughes, 2006). The cooperation of various motifs enables ATP binding and hydrolysis in NLRP3, represented by NACHT, LRR, and PYD. NACHT is further divided into four domains: a nucleotide-binding domain (NBD), a helix domain (HD1), a winged helix domain (WHD), and a second helix domain (HD2) (Dekker et al., 2021) (Figure 7).

The ATP-binding and hydrolysis-related motifs in NACHT NBD are the Walker A and Walker B motifs, respectively (Coll et al., 2019). The Walker A motif includes a highly conserved P-loop sequence, GXXGXGK (S/T), featuring the Lys232 residue that stabilizes interactions with the nucleotide’s terminal γ-phosphate. The Walker B motif, identified by the sequence DGX (D/E) E, includes acidic residues like Asp302 that coordinate Mg2+ ion binding and facilitate ATP hydrolysis (Sandall and MacDonald, 2019). In its inactive state, the ATP-binding site holds ADP, maintaining the inactive conformation (Sandall et al., 2020). Activation occurs when NLRP3 binds NEK7, allowing ATP to replace ADP and promote oligomerization. Inhibitors such as CY-09 and MCC950 target these motifs to prevent ATP hydrolysis and subsequent NLRP3 activation. CY-09 binds to the Walker A motif, while MCC950 interacts with the Walker B motif, demonstrating that these sites are critical for NLRP3 activation (Baron et al., 2015). Therefore, targeting the Walker A and Walker B motifs presents a promising approach for developing new NLRP3 inflammasome inhibitors.

### 6.2 Targeting ASC

In 1999, Masumoto et al. (Masumoto et al., 1999) discovered an ASC molecule with a molecular weight of 22 kDa in leukemia cells. Subsequent studies have demonstrated that ASC is a crucial component of the NLRP3 inflammasome, playing a significant role in dual capacities. ASC is recruited at the N-terminal PYD of NLRP3 to form ASC spots, while also recruiting Pro-Caspase-1 through the isotypic reaction at the C-terminal CARD. These ASC spots are oligomers formed through either CARD-CARD or PYD-PYD homologous protein interactions, which induce the cleavage and activation of Pro-Caspase-1 (Fernandes-Alnemri et al., 2007). Given the two-domain structure of ASC, PYD, and CARD, rational drug design appears highly feasible. Recently, Wang et al. (Wang et al., 2023) identified Atranorin, a lichen-derived secondary metabolite, as a novel inhibitor of NLRP3 targeting ASC proteins. Atranorin directly binds to ASC, inhibiting the oligomerization of ASC, NLRP3, and Pro-Caspase-1. Additionally, the small molecule ASC inhibitor MM01 shows an inhibitory effect on NLRP3 and other inflammasome sensors. These findings highlight the strong targeting potential of ASC for developing new and efficient NLRP3 inflammasome inhibitors. It is worth mentioning that, in addition to small molecule inhibitors, ASC-targeting nanoantibodies are also exciting as a new biologic treatment for inflammatory diseases caused by inflammasomes (Adriouch and Pelegrin, 2022). In this regard, the most notable study was the specific anti-ASC nanoantibody VHHASC reported by Bertheloot et al., and demonstrated its efficacy in breaking down already-formed ASC oligomers and treating inflammatory diseases in animal models (Bertheloot et al., 2022). This undoubtedly provides a new strategy for targeting the NLRP3 inflammasome against ASC.

## 7 Conclusion and prospect

With increasing evidence, AS is considered a chronic inflammatory vascular disease driven by various risk factors. The inflammatory response provides a comprehensive perspective to understand the complex pathogenesis of AS. As a signal transducer in the human immune system, the NLRP3 inflammasome plays an indispensable role in defending against foreign antigen invasion and regulating internal damage. However, its excessive activation can release various inflammatory factors, leading to plasma membrane pores and pyroptosis. Therefore, it is critical to recognize that aberrant NLRP3 activation is central to the occurrence and development of chronic inflammation in AS. Currently, there are various drugs for AS, each with limitations. Since AS is a chronic inflammatory disease, targeting the NLRP3 inflammasome pathway could represent an attractive new treatment route. As researchers have revealed the three activation pathways of the NLRP3 inflammasome, they are actively exploring related pharmacological interventions, focusing on two directions: 1) regulation of NLRP3 protein transcription and translation, 2) regulation of NLRP3 inflammasome initiation and activation. Current strategies for regulating NLRP3 transcription and translation include miRNAs, ubiquitination, and phosphorylation. Strategies for regulating the initiation and activation of the NLRP3 inflammasome include autophagy inducers and small molecule inducers. Among these, targeting the NLRP3 inflammasome with small molecule chemical inhibitors is a direct and ideal method, with potentially better efficacy better pharmacokinetics, and ease of use.

By analyzing the three components of the NLRP3 inflammasome and their potential binding sites, we found that the ATP binding site Walker A and ATP hydrolysis site Walker B in the NACHT domain of NLRP3 are crucial for its activation and represent potential drug binding sites. Combined with the configurations of classical and recent inhibitors specifically targeting NLRP3, MCC950, and CY-09 remain excellent leading compounds and deserve further modification. ASC, with only two domains of PYD and CARD, acts as an adapter in NLRP3 inflammasome assembly. MM01, a recently discovered broad-spectrum inflammasome inhibitor, shows significant ASC-targeted inhibition. Although its specific binding sites need further study, since ASC binds NLRP3 and Pro-caspase-1 through isotypic reactions, targeting ASC not only limits its function but also has the potential to attenuate the function of NLRP3 and Pro-caspase1. Therefore, the compound MM01 and nano antibody VHHASC as a leading drug design is very promising. Furthermore, as we mentioned earlier, given that dTGH was found to have a key role in NLRP3 inflammasome activation. Targeting dTGH may also be a very valuable direction for future NLRP3 inflammasome inhibition strategies. Current challenges in targeting the NLRP3 inflammasome as a treatment strategy for AS include: 1) Although both classic and latest small molecule inhibitors of the NLRP3 inflammasome have shown good *in vitro* results, clinical studies have been unsatisfactory. For example, MCC950 was discontinued in treating rheumatoid arthritis due to safety issues in a phase II clinical trial. Therefore, drug development based on existing small molecule inhibitors requires a series of clinical trials to verify the efficacy and safety in humans, which is a key step in translating laboratory results into clinical applications. 2) The need to ensure that the small-molecule inhibitor has high specificity and avoids affecting other inflammatory bodies or immune pathways. Meanwhile, long-term inhibition of the NLRP3 inflammasome may affect the body’s normal immune response and other physiological processes. Further studies on the efficacy and safety of long-term use are needed. 3) The occurrence and development of AS include multiple stages, and small molecule inhibitors may be more effective in specific stages. Therefore, determining the optimal therapeutic window and considering combinations with other agents may increase the complexity of the treatment strategy.

In any case, with the deepening understanding and analysis of chronic inflammation in atherosclerotic diseases, targeting the NLRP3 inflammasome has become a reasonable and effective emerging strategy for the treatment of AS, which will continue to promote the development of AS treatment.
